# Depression, Suicidality, and Substance Abuse Among Sexual Risk Behavior Subgroups of Male High School Students

**DOI:** 10.1007/s10508-026-03459-5

**Published:** 2026-05-17

**Authors:** Robert C. McMahon

**Affiliations:** Department of Educational and Psychological Studies, University of Miami, P.O. 248065, Coral Gables, FL 33123 USA

**Keywords:** Adolescent sexual risk behavior, Alcohol abuse, Suicidal behavior, Depression

## Abstract

This study identified sexual risk behavior subgroups among male adolescents who ever had sexual intercourse based on the presence or absence of early sexual intercourse, forced sexual intercourse, and four or more lifetime sexual partners (N = 1551). Subgroups were compared on rates of recent alcohol abuse, symptoms of depression, and suicide risk behaviors. Cluster analysis was used to derive sexual behavior subgroups and multinomial regression was used to compare subgroups on alcohol abuse and psychiatric symptoms. Substantially higher proportions reporting alcohol abuse, depressive symptoms, suicide planning, and attempts were associated with membership in a subgroup defined primarily by forced sexual intercourse, in contrast with a low-sexual-risk behavior reference group. Younger age and more suicide attempts were linked with membership in a cluster subgroup defined primarily by having experienced early sexual intercourse compared with the reference group. Finally, older age, more alcohol abuse, and suicide attempts were associated with membership in a subgroup defined by having had four or more lifetime sexual partners as opposed to the reference group. The cluster subgroups of sexually experienced male high school students varied significantly in proportions reporting early coitus, forced sexual intercourse, and four or more lifetime sexual partners, and appear to represent relatively distinct sexual risk profiles. Intervention implications associated with sexual risk behavior and mental health problems identified among sexually active male high-school students are discussed.

## Introduction

Analysis of trends in adolescent sexual behaviors and mental health status in the United States reveals meaningful change. Herbenick et al. (2022) report substantial declines in sexual activity among both male and female US adolescents (ages 14 through 17) between 2009 and 2018. Similarly, according to a decadal review of Youth Risk Behavior Survey (YRBS) data, in 2011, nearly half of female and male high school students reported having engaged in sexual intercourse, with approximately 15% indicating four or more sexual partners (CDC, 2021). In contrast, by 2021, fewer than one-third reported sexual intercourse, and only 6% had four or more partners. However, the incidence of forced sexual intercourse did not decline, particularly among young women. Four percent of male students reported experiencing forced sexual intercourse (FSI) in both 2011 and 2021, while the proportion for female students increased from 12% in 2011 to 14% in 2021. There have been substantial changes in rates of initiation of sexual activity, in the frequency of multiple sexual partnering, and perhaps in the degree of self-control in sexual activity decision-making. Herbenick et al. (2022) suggest that changes in adolescent sexual activity and in relationships involving sexual intimacy may influence important mental health outcomes. Interestingly, significant increases in depression and suicidality have been reported over the past decade among both males and females, with particularly substantial increases evident among female adolescents (CDC, 2021; Daly et al., 2022). Careful prospective studies have helped clarify the predictive significance of adolescent sexual behaviors in relation to physical and mental health outcomes (e.g., Guiney et al., 2024; Ramrakha et al., 2013; Vasilenko et al., 2015, 2016). Less is known about how traditionally emphasized patterns of adolescent sexual risk behavior (i.e., early coitus, forced intercourse, and multiple sexual partnering) are linked to depression, suicidal behaviors, and substance abuse among recent cohorts of adolescents.

### Forced Sexual Intercourse

Several investigations have evaluated the relationship between histories of forced sexual experiences and subsequent sexual risk behaviors, sexually transmitted infections (STIs), and psychiatric disorders among young men (Guiney et al., 2024; Smith & Ford, 2010). Smith and Ford, using data from 1400 males aged 18 to 24 sampled in the National Survey of Family Growth, identified significant associations between forced sexual contact and subsequent sexual risk behavior and STIs, with stronger associations when perpetrators were male. Both male and female perpetrators were reported to use verbal coercion, alcohol, and drugs. Additionally, an international birth cohort study demonstrated that reports of forced sexual activity, including incidents in childhood, were prospectively associated with internalizing and externalizing psychopathology, thought disorders, depression, and suicide attempts over extended periods (Guiney et al., 2024). Data from the YRBS (2001–2017; Pontes et al., 2022) indicated a strong association between FSI and depressive symptoms, suicidal ideation, suicide attempts, and medically treated suicide attempts across genders. While females reported higher rates of FSI and a stronger link with depressive symptoms, males who experienced FSI were more likely to report suicide attempts requiring medical intervention. Notably, this vulnerable subgroup of males remains underrepresented in research (Pontes et al., 2022).

### Early Sexual Intercourse

Early age at first sexual intercourse (ESI) is recognized as an important risk marker. Sex occurring at a very early age tends to be less consensual and protected (Abma & Martinez, 2023; Houts, 2005; Martinez et al., 2011; Osorio et al., 2017). Findings from the National Longitudinal Study of Adolescent Health (Add Health) demonstrate that early sexual initiation predicts increased sexual risk behavior, STIs, and pregnancy in young adulthood (Vasalinko, 2022). The Seattle Social Development Project found similar results, indicating that early sexual activity forecasts increased sexual risk behaviors in the twenties and thirties, including multiple partners, sex while intoxicated, inconsistent condom use, and involvement in prostitution. However, these effects are moderated by childhood behavioral disinhibition and peer delinquency. Further, Add Health data suggest that earlier onset of sexual behavior is associated with heightened depressive symptoms in young adulthood—a relationship influenced by gender and the nature of romantic relationships with sexual partners (Kugler et al., 2017; Mendle et al., 2016; Vasilenko et al., 2016).

There is evidence of both shared and independent contributions of early sexual initiation and forced sexual intercourse to mental health challenges. Baiden et al. (2021) used combined data from the 2017 and 2019 YRBS to analyze a sample of 6,252 adolescents aged 14 to 18. Within this group, 7.1% reported sexual initiation before age 13, and 14.8% reported experiencing FSI. After controlling for FSI, ESI was significantly correlated with suicidal ideation, planning, and attempts. Adjusting for ESI and demographics, FSI was associated with elevated risks of depression, suicidal ideation, planning, and attempts. Female adolescents with FSI demonstrated substantially higher risks of suicidal ideation, planning, and attempted suicide compared to males.

### Multiple Sexual Partners

Adolescents reporting multiple sexual partners present a public health concern due to potential links with both FSI and ESI, and heightened vulnerability to STIs. These patterns are linked with unintended pregnancies, potentially fertility-impairing STIs, and substance misuse (Epstein et al., 2014; Heywood et al., 2015). Cavazos-Rehg et al. (2009) analyzed YRBS data (1999–2007) to assess connections between substance use and the number of sexual partners. Increased substance use correlated with higher partner counts across genders. While multiple sexual partners are generally associated with substance abuse and depression among U.S. adolescents, these relationships tend to diminish in early adulthood (Epstein et al., 2014); persistent associations with depression are primarily observed among women (Vasilenko & Lanza, 2014). However, the Dunedin, New Zealand longitudinal cohort study found consistent prospective associations across genders between the number of sexual partners and subsequent anxiety, depression, and substance dependence disorder into young adulthood (Ramrakha et al., 2016). Kahn and Halpern (2018), drawing on a subset of 6,587 respondents from the Add Health study, determined that fewer lifetime sexual partners were associated with lower odds of STI/STD diagnosis, unintended pregnancy, and improved relationship quality. Interestingly, these associations did not apply to individuals whose average age of sexual onset was 22 years.

### Study Aims

An understanding of how patterns of early onset sexual intercourse, forced sexual intercourse, and sexual intercourse with multiple sexual partners may cluster among certain groups and how such groups may differ on important mental health indicators can inform public health strategies to mitigate risk and promote protective behaviors. Howard and Hoffman (2018) recommend person-centered methodologies to identify optimal subpopulations, allowing accurate sample representation based on key parameters, with cluster analysis serving as a pivotal tool. McMahon (2025) identified sexual risk cluster subgroups among sexually active U.S. female adolescents (2021 CDC YRBS) according to the presence or absence of ESI, FSI, and four or more lifetime sexual partners (≥ 4 LSP). Elevated rates of alcohol abuse, depressive symptoms, and suicide attempts were noted among those in clusters defined by 4 or more lifetime sexual partners and by forced sexual intercourse relative to a sexually active low-risk reference group. Higher proportions revealing suicide contemplation and attempts were found in a cluster characterized by both early sexual initiation and forced sexual intercourse compared to the low-risk reference group. The present study extends this work by: (1) identifying sexual risk behavior cluster subgroups among US male high school students who reported ever having sexual intercourse, using data from the 2021 YRBS capturing early onset sexual intercourse (ESI), forced sexual intercourse (FSI), and 4 or more lifetime sexual partners and (2) comparing sexual risk cluster subgroups on recent alcohol abuse, depressive symptoms within the last year, and suicide-related behaviors. It was hypothesized that clusters defined by the prominence of one or more sexual risk behaviors would report higher levels of substance dependence, depression, and suicidal behaviors than a lower risk cluster subgroup.

## Method

### Participants and Procedure

This study used data from the 2021 Youth Risk Behavior Survey (YRBS), a biennial, school-based national survey conducted by the Centers for Disease Control and Prevention (CDC), to examine adolescent health-risk behaviors contributing to leading causes of death and disability in the United States (YRBS Data Users Guide, 2021). Employing a three-stage cluster sampling design, the survey team recruited 9th through 12th graders from public and private schools and used self-administered questionnaires. Comprehensive details on objectives, methodology, sampling procedures, and IRB approval are available in referenced publications (Mpofu, 2023). In 2021, the national YRBS sample comprised 17,232 high school students, including 8816 males; 1956 (22.18%) reported ever having had sexual intercourse, with analysis restricted to 1551 males providing complete data on the three sexual risk variables used for clustering.

### Measures

#### Subgrouping Variables

Adolescents who reported never having had sexual intercourse were excluded from analysis. The primary grouping variables were ESI, history of FSI, and lifetime experience of four or more sexual partners (≥ 4 LSP). ESI was determined by the response to “How old were you when you had sexual intercourse for the first time?” Those indicating first intercourse before age 13 were coded as “1”; first intercourse at age 13 or older was coded as “0”. FSI was indicated by an affirmative answer to “Have you ever been forced to have sexual intercourse when you didn’t want to?”, coded as “1” for Yes and “0” otherwise. The lifetime number of sexual partners was coded “1” for those reporting four or more partners and “0” for fewer than four.

#### Mental Health Variables

Depressive symptoms were classified based on respondents affirming persistent sadness or hopelessness for at least two weeks in the prior year, resulting in cessation of usual activities (coded “1” for yes, “0” for no). Suicidal ideation reflected serious consideration of suicide in the past 12 months (“1” for yes, “0” for no). Suicide planning and attempts were similarly coded using the YRBS protocol. Alcohol abuse was assessed by reported consumption of five or more drinks in succession on at least one day in the past thirty days (“1” for yes, “0” for no).

#### Control Variables

Demographic controls included age (five levels: ≤ 14, 15, 16, 17, ≥ 18), race/ethnicity (American Indian, Asian, Black/African American, Hawaiian/Pacific Islander, White, Hispanic/Latino, Multiethnic-Hispanic, Multiethnic Non-Hispanic), and gender of sexual partners (female only, male only, both), using mutually exclusive coding (1 for category endorsement, otherwise 0).

### Data Analyses

Data was initially analyzed using descriptive statistics and two-step cluster analysis. The clustering procedure grouped participants by sexual risk behaviors, considering binary indicators: ESI, FSI, and having four or more lifetime sexual partners (4 + LSP); temporal sequence of risk behaviors was not considered. Log-likelihood measured distance between cases, and the Bayesian Information Criterion (BIC) identified the optimal number of clusters by balancing fit and simplicity. Multinomial logistic regression was employed to assess cluster subgroup associations with substance abuse and mental health status, adjusting for covariates.

## Results

### Sample Characteristics

The 2021 national YRBS included 17,232 high school students, of whom 8816 were male. Of these males, 1,956 reported having engaged in sexual intercourse. Binary indicators for missingness were created for forced sexual intercourse, early sexual intercourse, and having four or more lifetime sexual partners; these indicators were analyzed in relation to five measures reflecting depressive, suicidal, and alcohol abuse symptoms. The correlations between the missingness indicators for sexual risk behaviors and mood, suicidal, and substance use symptoms ranged from *r* = .004 to .086, indicating minimal association. Analysis focused on the subset of 1,551 respondents who completed all three sexual risk variable responses used to form cluster subgroups. Among these 1,551 adolescents who had sexual intercourse, 11.5% reported early sexual initiation (first intercourse before age 13), 8.9% reported experiencing forced sexual intercourse, and 24.7% reported having had four or more sexual partners. Regarding racial and ethnic self-identification, 45.4% identified as White, 16.7% as Black or African American, 6.6% as Hispanic/Latino, 11% as Multiethnic-Hispanic, 6.6% as Asian, 1.4% as American Indian or Alaskan Native, and 1.3% as Hawaiian or Pacific Islander. 89.3% reported female-only sexual partners, 3.7% male-only partners, and 4.4% both female and male partners. Approximately 41% indicated feeling so sad or hopeless nearly every day for at least two consecutive weeks during the past year that they ceased participating in usual activities. Furthermore, 23.3% reported suicidal ideation, 19.6% reported having made a suicide plan, 11.8% reported attempting suicide, and 3.7% reported an attempt necessitating medical intervention. Regarding substance use, 21.8% reported at least one day in the past 30 days when they consumed five or more drinks consecutively. Table 2 provides details on sexual risk, racial/ethnic categories, sexual partner male versus female status, depression/suicidality, and substance abuse status by cluster subgroup.

### Cluster Subgroup Analysis

A two-step cluster analysis was conducted utilizing the Youth Risk Behavior Surveillance Survey (YRBS) data from male U.S. high school students who reported lifetime sexual intercourse. The analysis incorporated variables for first sexual intercourse before age 13 (ESI), being physically forced into unwanted sexual intercourse (FSI), and having had intercourse with four or more lifetime partners (4 or more LSP). A silhouette plot analysis assessed the fit of data points within clusters, with results supporting a four-cluster solution (Fit Index > .9) reflecting strong cohesion and separation. Refer to Table 1 for further details. Chi-square tests were performed to evaluate differences among cluster subgroups based on YRBS responses concerning recent binge drinking (χ^2^ = 62.61; df = 3, *p* < .001), sadness/hopelessness (χ^2^ = 84.07; df = 3, *p* < .001), serious consideration of suicide (χ^2^ = 135.39; df = 3, *p* < .001), suicide planning (χ^2^ = 125.68; df = 3, *p* < .001), suicide attempts (χ^2^ = 184.66; df = 3,* p* < .001), and suicide attempts resulting in injury or illness requiring medical attention (χ^2^ = 98.71; df = 3, *p* < .001). Table 2 shows percentages by age group, race/ethnicity, male/female status of intimate partners, recent alcohol abuse, and psychiatric symptom status by Cluster Subgroup.

**Table 1:** 2-step cluster analysis proportions reporting each sex risk behavior by cluster subgroup

Sexual risk behavior subgroups	Cluster 1 n = 1036 Low risk	Cluster 2 n = 138 Forced sexual intercourse	Cluster 3 n = 127 Early sexual intercourse	Cluster 4 n = 250 Four or more sexual partners
*Variables*				
Forced	0	100	0	0
Sexual				
Intercourse				
Early	0	37.0	100	0
Sexual				
Intercourse				
> 3 Life	0	47.1	53.5	100
Sexual				
Partners

**Table 2:** Demographics, partner characteristics, alcohol abuse, depression, and suicidal behavior by cluster subgroup

Variables: All values reported as percentages	Cluster 1 Low risk reference n = 1036	Cluster 2 Forced sexual intercourse n = 138	Cluster 3 Early sexual intercourse n = 127	Cluster 4 Four or more sexual partners n = 250	Total	χ^2^ (df) *p*-Value
*Sexual risk behavior subgroups*						
Age						98.23 (21), *p* < 0.001
14 or Younger	7.6	12.3	22.1	4.4	8.5	
15	16.5	11.6	24.4	10.4	15.7	
16	28.0	31.9	23.6	20.4	26.8	
17	35.5	31.2	23.6	42.8	35.3	
18 or Older	12.4	13.0	6.3	22.0	13.5	
Race/Ethnicity						
Am. Indian/	1.4	2.2	.8	.8	1.4	1.64 (3), *p* = .65
Asian	7.1	8.0	2.4	5.6	6.6	5.03 (3), *p* = .16
Black/African American	15.8	15.9	15.0	21.6	16.7	5.21 (3), *p* = .15
Hawaiian/Pacific Islander	1.1	1.4	.8	2.4	1.3	3.12 (3), *p* = .37
White	46.8	41.3	46.5	41.2	45.4	3.60 (3), *p* = .30
Hispanic/Latino	6.9	4.3	6.3	7.2	6.6	1.39 (3), *p* = .70
Multiethnic-Hispanic	10.1	13.8	17.3	9.6	11.0	7.58 (3), *p* = .06
Multiethnic Non-Hispanic	7.6	10.1	8.7	7.6	7.9	1.18 (3), *p* = .75
(Sexual Partners)						
Female only	92.4	59.6	86.8	94.4	89.3	190.55 (3) *p* < .001
Men only	2.9	10.7	2.6	3.7	3.7	27.35 (3) *p* < .001
Male and Female	1.9	27.7	6.0	1.7	4.4	242.96 (3) *p* < .001
Alcohol Use						
> 4 alcoholic drinks last 30 days	16.8	39.6	17.6	36.4	21.8	62.61 (3), *p* < 0.001
Psychiatric status						
Depressive symptoms	38.0	78.5	43.2	36.2	41.7	84.07 (3), *p* < 0.001
Suicidal ideation	19.3	63.7	21.6	18.6	23.3	135.39 (3), *p* < 0.001
Suicide planning	15.7	57.4	24.0	14.2	19.6	125.68 (3), *p* < 0.001
Suicide attempt	6.9	48.0	16.7	8.9	11.8	184.66 (3) *p* < 0.001
Suicide attempt with treatment	1.8	20.7	3.3	2.4	3.7	98.71 (3), *p* < .001

Cluster 1 (Low Sexual Risk): None of the 1036 males reported a history of forced sex, early sexual activity, or 4 + partners. 16.8% reported recent binge drinking. Rates for sadness/hopelessness, suicide consideration, planning, attempts, and serious attempts were 38%, 19.3%, 15.7%, 6.9%, and 1.8%, respectively.

Cluster 2 (Forced Sexual Intercourse): All 138 males had experienced forced sex; 37.0% reported early sex, 47.1% had 4 or more partners, and 39.6% reported binge drinking. Sadness/hopelessness was reported by 78.5%, suicide consideration by 63.7%, planning by 57.4%, attempts by 48.0%, and serious attempts by 20.7%.

Cluster 3 (Early Sexual Intercourse): All 127 reported early sex but not forced sex; 53.5% had 4 or more partners, and 17.6% had a binge episode. Rates for sadness/hopelessness, suicide consideration, planning, attempts, and serious attempts were 43.2%, 21.6%, 24.0%, 16.7%, and 3.3%.

Cluster 4 (4 + Sexual Partners): All 250 had 4 or more partners but no early or forced sex; 36.4% reported alcohol abuse. Sadness/hopelessness, suicide consideration, planning, attempts, and serious attempts were 36.2%, 18.6%, 14.2%, 8.9%, and 2.4%.

### Multinomial Regression

Multinomial logistic regression was applied to examine whether factors such as age, race/ethnicity, recent alcohol abuse (binge drinking), depression, suicidal thoughts, suicide planning, suicide attempts, and attempts requiring medical intervention were linked to membership in three clusters of male high school students engaged in elevated sexual risk behavior relative to a lower sexual risk behavior reference group. These cluster comparisons included: Cluster 2 (Forced Intercourse) versus the low-risk reference group (Cluster 1); Cluster 3 (Early Sexual Intercourse) versus the reference, and Cluster 4 (Four or More Sexual Partners) versus the reference. The statistical model showed significant results (Final Model Fitting Criteria AIC = 1442; 2-Log Likelihood = 362.39; χ^2^ = 362.39, df = 57, *p* < .001; Nagelkerke R^2^ = .25).

After controlling for the effects of age and race/ethnicity, higher proportions reporting alcohol abuse (B = .73; SE = .23; Wald = 10.08, df = 1, *p* = .002, Exp(B) = 2.09), depressive symptoms (B = .65; SE = .27; Wald = 5.68, df = 1, *p* = .017, Exp(B) = 1.91), suicide contemplation (B = .79; SE = .31; Wald = 6.44, df = 1, *p* = .011, Exp(B) = 2.20), and suicide plan (B = .93; SE = .31; Wald = 8.63, df = 1, *p* = .003, Exp(B) = 2.53) were associated with membership in Cluster 2 (Forced Sex Intercourse) in contrast with the Low Sexual Risk Reference (Cluster 1). Younger age (Age 14: B = 1.53, SE = .51; Wald = 8.83, df = 1, *p* < .003, Exp(B) = 4.62; and Age 15: B = 1.21; SE = .49; Wald = 6.08; df = 1; *p* = .014; Exp(B) = 3.35) and more suicide attempts (B = 1.56; SE = .46; Wald = 11.29, df = 1,* p* < .001), Exp(B) = 4.78) were linked with membership in Cluster 3 (Early Sexual Intercourse) compared with the Cluster 1 reference group. Finally, after controlling for the effects of age and race/ethnicity, more suicide attempts (B = .92; SE = .39; Wald = 5.47, df = 1, *p* = .019, Exp(B) = 2.51) and more recent alcohol abuse (binge drinking) (B = .99; SE = .16; Wald = 37.13, df = 1, *p* < .001, Exp(B) = 2.70) but few suicide attempts requiring treatment (B = -2.38; SE = 1.04; Wald = 5.27, df = 1, *p* = .022, Exp(B) = .09) were associated with membership in Cluster 4 (4 or More Lifetime Sexual Partners) as opposed to the Cluster 1 Reference group.

## Discussion

This study identified 4 sexual behavior cluster subgroups of male high school students who reported any lifetime sexual intercourse and compared these groups on rates of alcohol abuse, depressive symptoms, and suicide risk indicators on the 2021 YRBS. In the largest cluster subgroup (Cluster 1, Low-Sexual Risk), no participant indicated having 4 or more lifetime sexual partners; most reported one (62%) or two (25%) lifetime partners; none reported very early onset sexual activity (ESI) or forced sexual intercourse (FSI). However, moderate rates of recent substance abuse, depression during the past year, and suicidal behavior were reported in this comparatively low sexual risk group.

In Cluster 2 (Forced Sexual Intercourse), all members reported FSI, more than a third reported ESI, and nearly half reported 4 or more lifetime sexual partners (4 or more LSP). About 6 in 10 reported sexual activity only with women, about 11% only with men, and more than a quarter with men and women. Nearly 80% reported past-year depression, and substantial numbers reported suicide attempts and recent alcohol abuse. This pattern raises concern about self-injurious behavior and unsafe sexual practices that warrant evaluation and intervention planning. Although it is not clear what proportion of those reporting forced intercourse could be characterized as having experienced sexual trauma, reports from earlier YRBS surveys have linked forced intercourse to high levels of depression, suicidal ideation, suicide attempts, and, among males, suicide attempts requiring medical attention (Pontes et al., 2022). Regarding longer-term implications of early sexual abuse, a recently published 5-decade follow-up analysis of the Dunedin, New Zealand, longitudinal birth cohort reveals that “survivors” were more likely than their peers to experience a broad range of psychological disturbances, suicide attempts, sexually transmitted diseases, conflictual relationships, and antisocial behavior, among other problems (Guiney, et al., 2024). They evaluated the risk of adverse outcomes in multiple life domains across adulthood.

Pontes et al. (2022) identified several factors that have likely contributed to the lack of focus on male FSI. These include reluctance to take male FSI reports seriously, particularly when females are reported perpetrators. Victim blaming is evident and may be due to the presumption that sexual victimization is best understood as involving a dominating predator against an unsuccessfully resisting vulnerable party**.** Oliffe and Phillips (2008) argue that there have been difficulties overcoming biases involving masculine self-reliance and minimization of vulnerability and distress on the part of victims as well as among potential healthcare providers. Research is gaining momentum to demonstrate the crucial need to better prevent sexual victimization at all levels: family, school, and the community at large (Javaid, 2016).

All members of Cluster 3 (Early Sexual Intercourse) reported experiencing their first sexual intercourse before age 13 (ESI); however, none reported FSI. More than half reported 4 or more lifetime sexual partners, and more than a third reported having 6 or more. Depressive symptoms during the past year were reported by 43%, and nearly a quarter reported having planned a suicide attempt during this period. Current findings are consistent with those of Baiden et al. (2021), who found that after controlling for the effects of FSI, early sexual intercourse (ESI) was significantly associated with suicidal ideation, suicide planning, and suicide attempts. A rather extensive literature has identified linkages between ESI and the number of lifetime sexual partners, sexual activity under the influence of drugs, sexually transmitted infections, pregnancy, births, and abortion among adolescents (Heywood et al., 2015; Kugler et al., 2017; Sandfort et al., 2001; Vasilenko et al., 2016). Epstein et al. (2014) found that early sexual activity predicts sexual risk behavior in young adulthood (20s and early 30s) and that the number of lifetime partners explained the link between early sexual intercourse and sexually transmitted infection. ESI has also been linked with adolescent and parental substance use, aggression, conduct disorder, difficulties with family attachment and living situations, school achievement difficulties, and limited maternal education (Reis et al., 2023).

The Cluster 4 (Four or More Lifetime Sexual Partners) subgroup consisted of 250 males reporting no history of early sexual intercourse or forced intercourse. However, more than 40% reported at least 6 lifetime partners. As expected, the proportion of males reporting 4 or more lifetime sexual partners was higher among older students. A quarter of this cluster subgroup were high school juniors, and more than half were seniors. The moderate levels of depression and suicide risk behavior that were reported in this subgroup were marginally higher compared to those in the Low-Sexual-Risk reference group. However, higher levels of alcohol abuse were reported compared to the Reference group. Cavazos-Reg et al. (2009) used YRBS data aggregated from 1999 to 2007 in the examination of linkages between initiation and intensity of substance use and the number of sexual partners. For both male and female students, the number of sexual partners rose consistently as substance use shifted from no use to early experimentation, to heavy use. A potentially concerning aspect of the linkage between having more partners and greater substance abuse emerges in the Dunedin, New Zealand longitudinal birth cohort study. Ramrakha et al. (2016) found that across 3 young adult age groups, having more sexual partners predicted increased substance abuse diagnosis.

### Implications

The cluster subgroups of sexually experienced male high school students differed substantially in proportions reporting early coitus, forced sexual intercourse, and multiple lifetime partners. These clusters appear to reflect relatively distinct sexual risk profiles. 3 of the 4 subgroups represent young men with significant sexual health and mental health concerns. Despite meaningful differences in sex risk behavior, one subgroup was defined by having 4 or more lifetime sexual partners, and about half in the Forced Sexual Intercourse and Early Sexual Intercourse clusters also reported this pattern. Grubb and Powers (2020) argue that practices for addressing the reproductive and sexual health needs of young men are not as well established as those for young women. Primary care physicians are substantially less likely to gather sexual health information from young men or to counsel on barrier protection methods. They present updated guidelines relating to male adolescents’ sexual and reproductive health that include having discussions regarding the consequences of sexual activity, consent for sexual acts among adolescents, their roles in contraception decision-making, STIs and STI screening and treatment, human papillomavirus vaccine, and information on sexual dysfunction among adolescent and young adult males.

Across cluster subgroups, meaningful numbers reported recent alcohol abuse, as well as depression, suicidal ideation, suicide planning and attempts over the past year. The US Preventive Services Task Force recommends depression screening for adolescents aged 12–18, as MDD is a major risk factor for suicide attempts and completion among US youth aged 10 to 19 years. If the suicide risk is high, timely implementation of care is crucial (Treatment for Suicidal Ideation, Self-harm, and Suicide Attempts Among Youth. SAMHSA, 2020). Substance abuse also contributes significantly to risk status, with substantial binge drinking reported across cluster subgroups in the current investigation. Screening, Brief Intervention, and Referral to Treatment is a universal intervention used to evaluate substance use severity and level of indicated treatment. When abuse severity is high, referral to comprehensive, evidence-based care is essential (Walter et al., 2023).

### Limitations

Because the study was cross-sectional, it cannot determine causality between sexual risk events and mental health outcomes. Symptoms of depression, suicidality, and substance abuse might have preceded, followed, or coincided with sexually risky behaviors. Data relies on self-reports, which may be influenced by recall bias or social desirability, and varying timeframes could affect reliability. The social-relational context of sexual risk behavior is complex and may influence responses to items gauging mental health symptom status. Reports regarding suicidal ideation, planning, and attempts by substantial numbers of sexually active male adolescents raise concerns about well-being but also about response reliability and validity. While the YRBS offers broad estimates of adolescent risk status, more robust screening measures would improve assessment and facilitate appropriate safeguards and care.

### Conclusions

This research examined patterns of sexual risk behavior in a nationally representative sample of male high school students. Three cluster subgroups defined by the presence of early sexual intercourse, forced sexual intercourse, or 4 or more lifetime sexual partners were compared with a low-sexual-risk reference group on 4 indicators of mental health status. These cluster subgroups of sexually experienced male high school students varied significantly in proportions reporting sexual risk behavior and may represent distinct profile types. Evaluations to address sexual risk behavior and mental health concerns identified among sexually active male high school students are recommended.

## Data Availability

National- and site-level 2021 YRBS data are available in a combined dataset from the YRBSS data and documentation website (https://www.cdc.gov/healthyyouth/data/yrbs/data.htm).

## Code Availability

Coding documentation is available on the Centers for Disease Control’s Data & Documentation page for the 2021 National YRBS results (https://www.cdc.gov/healthyyouth/data/yrbs/data.htm).
